# Emerging Shiga-toxin-producing *Escherichia coli* serogroup O80 associated hemolytic and uremic syndrome in France, 2013-2016: Differences with other serogroups

**DOI:** 10.1371/journal.pone.0207492

**Published:** 2018-11-12

**Authors:** Brecht Ingelbeen, Mathias Bruyand, Patricia Mariani-Kurkjian, Simon Le Hello, Kostas Danis, Cécile Sommen, Stéphane Bonacorsi, Henriette de Valk

**Affiliations:** 1 Santé Publique France, Saint-Maurice, France; 2 European Programme for Intervention Epidemiology Training (EPIET), European Centre for Disease Prevention and Control (ECDC), Stockholm, Sweden; 3 Service de Microbiologie, Centre National de Référence associé, Centre Hospitalo-Universitaire Robert-Debré, AP-HP, Paris, France; 4 IAME, UMR 1137, INSERM, Université Paris Diderot Sorbonne Paris Cité, Paris, France; 5 Institut Pasteur, Paris, France; Istituto Di Ricerche Farmacologiche Mario Negri, ITALY

## Abstract

To generate hypotheses on possible sources of Shiga toxin-producing *Escherichia coli* (STEC) serogroup O80 associated hemolytic-uremic syndrome (HUS), we explored differences in factors associated with STEC O80 associated HUS, compared with STEC O157 or STEC of other serogroups, in France during 2013–16. STEC was isolated from 153/521 (30%) reported HUS cases: 45 serogroup O80, 46 O157 and 62 other serogroups. Median ages were 1.1 years, 4.0 years and 1.8 years, respectively. O80 infected patients were less likely to report ground beef consumption (aOR [adjusted Odds Ratio] 0.14 95% CI [Confidence Interval] 0.02–0.80) or previous contact with a person with diarrhea or HUS (aOR 0.13 95%CI 0.02–0.78) than patients infected with STEC O157. They were also less likely to report previous contact with a person presenting with diarrhea/HUS than patients infected with other serogroups (aOR 0.13 95%CI 0.02–0.78). STEC O80 spread all over France among young children less exposed to known risk factors of O157 or other STEC infections, suggesting the existence of different reservoirs and transmission patterns.

## Introduction

Shiga-toxin-producing *Escherichia coli* (STEC) infections can result in gastroenteritis, enterocolitis, and bloody diarrhea [1]. In up to 10% of STEC infections hemolytic-uremic syndrome (HUS) may develop, making STEC the main cause of acute renal failure in children [1]. Humans can contract STEC infection through consumption of food items contaminated by the animal reservoir such as undercooked beef, raw milk, or raw vegetables, through contact with animals or an environment contaminated by colonized animals, or through person-to-person contact in particular in nurseries or within families [1–6]. STEC serogroup O157 infection has been best described, with cattle as the main reservoir among several other mammals and birds [7–9].

Since 2005, STEC serogroup O80 has emerged to become in 2015 a predominant serogroup among pediatric HUS patients reported in France, along with the STEC O157 and O26 serogroups. Patients infected with STEC O80 may develop specific clinical features such as septicemia [10]. Also in Switzerland human STEC O80 infections were reported between 2010 and 17, with 72% in regions that share borders with France, and 33% ≤5 years of age. One case developed HUS [11]. The reservoir of STEC O80 remains unknown. Identifying the reservoir and the source of infection would allow the implementation of targeted prevention and control measures for STEC O80 infections.

We described the geographical and seasonal distribution, characteristics and exposures of STEC O80 pediatric HUS cases detected by the French surveillance system and compared them with other STEC serogroups, in order to generate hypotheses on potential sources of infection or reservoirs.

## Methods

We compared STEC O80 cases with other STEC serogroups (case-case study) amongst children infected with STEC that developed HUS (STEC associated HUS), reported to the French pediatric HUS surveillance system between 2013 and 2016. The HUS pediatric surveillance system comprises thirty-two pediatric nephrology departments located in public hospitals evenly distributed throughout France that report all HUS cases in children to the French public health agency.

### Case definitions, inclusion and exclusion criteria

Within the surveillance system, HUS was defined as acute hemolytic anemia (hemoglobin <10 g/100 mL or red blood cell fragments ≥ 2%) and acute renal failure (serum creatinine level >60 μmol/L if age <2 years, >70 μmol/L if age ≥2 years) [12]. Only HUS patients aged below 15 years and without travel history in the 7 days before the onset of diarrhea were reported. Stool samples and isolates were sent to the National Reference Laboratory (Centre Hospitalo-Universitaire Robert-Debré, Paris, France) where the STEC serogroup was determined using polymerase gene *wzy*-specific PCR or detection of O antigen genes cluster *rfb* restriction fragments length polymorphism on an isolated strain [10].

In our study, we defined as cases, HUS patients reported to the HUS surveillance between 2013 and 2016, for whom STEC was isolated and a serogroup identified. We excluded cases with stool cultures yielding multiple serogroups. Of cases belonging to the same outbreak, i.e. an increase in the number of cases for which there is a strong hypothesis of common exposure or person to person transmission, we included only the case with the earliest onset of symptoms.

### Data collection

For any reported HUS, a case report recorded patients’ demographics and main exposures in the 15 days preceding the symptom onset: consumption of food items known to be associated with STEC infections, contact with a diarrhea or HUS case, attending nurseries, kindergartens or schools, and contact with animals. Depending on the health facility reporting the HUS, a food questionnaire documenting food items consumed in the 7 days before symptom onset (14 days if STEC serogroup O104) was also completed. In 2015, following an increase in STEC O80 infections, STEC O80 infected HUS patients were systematically interviewed. We used the same food questionnaire, with additional questions on travel history, contact with animals, exposure to water, and type of housing. HUS case reports, food questionnaires with and without additional exposure questions were paper-based and entered into an electronic database.

### Data analysis

We compared characteristics and exposures of patients infected with STEC O80 with those of patients infected with STEC O157, and with STEC serogroups other than O80 or O157.

We described the temporal and spatial distribution and characteristics of patients infected with STEC O80, STEC O157 and STEC of other serogroups. We calculated annual incidence per region of the patients’ residence, for STEC O80, STEC O157 and STEC cases of other serogroups. We compared frequencies (with proportions) and medians (with interquartile ranges (IQR)) of patient characteristics between STEC O80 and STEC O157, or STEC of other serogroups. We used Pearson’s Chi-squared test and Fisher’s exact test, as appropriate, to compare frequencies, and the Wilcoxon-Mann-Whitney U test to compare medians. We considered differences or associations at the 5 percent level statistically significant. We estimated seasonality of STEC O80 and compared it with seasonality of STEC O157 using time series Poisson regression including a sine and cosine curve for a 12 month period. We used a Wald test to compare the timing of the seasonal peaks between the serogroups. To identify the potential differences between exposures of different serogroup STEC infections, we used unconditional logistic regression to yield crude odds ratios (with 95% confidence intervals (CI)) for all exposure variables. Exposures that were documented for all study patients (urban residency, contact with a person with diarrhea or HUS, attending nursery or school, contact with farm animals, any swimming, and consuming ground beef steak, raw milk, or cheese from raw milk) and that were associated with STEC O80 infection by univariate analysis (p < 0.1) were included in a logistic regression model. We used stepwise deletion of exposure variables on the basis of statistical and epidemiological criteria to obtain the final multivariable regression model. We calculated adjusted odds rations with 95% CI for the exposures that were retained in the final model. We included age as a continuous variable, using a multivariable fractional polynomial model. We used STATA 12 (StataCorp LP, College Station, TX, USA) to analyze the data and QGIS 2.18 (Open Source Geospatial Foundation, Beaverton, OR, USA) with region shapefiles from http://data.gouv.fr, to generate choropleth maps, projected in Lambert-93.

### Ethics

Data were collected as part of the HUS surveillance led by the French public health Agency. The French law compels the health professionals to give, in conditions approved by the French data protection Authority (Commission Nationale Informatique et Libertés), the data required by the public health agency. The 4 year data used for the study were collected retrospectively and did not contain any patient-level identifying information. No formal approval from an ethical committee or informed consent was needed for the study presented.

No specific funding was required for this study.

## Results

From 1 January 2013 to 31 December 2016, 521 HUS cases were reported to the French pediatric HUS surveillance. STEC was isolated in 156 (30%) cases. We excluded one case with multiple strains isolated. Two small outbreaks of two cases each were reported: twice siblings with the same exposure and symptom onset on the same day. Only one of each pair of siblings was included, picked at random. 153 HUS cases with STEC infection were included in the study: 45 with STEC O80, 46 with STEC O157 and 62 with STEC of any other serogroup (36 O26; 7 O55; 6 O121; 5 O111; 4 O145; 1 O103; 1 O104; 1 O115; 1 O86). Food questionnaires were completed for 34 STEC O80, 42 STEC O157, and 52 STEC of other serogroups. Additional exploratory questions were completed for 11 STEC O80, 15 STEC O157 and 19 STEC of other serogroups.

**Fig 1:**
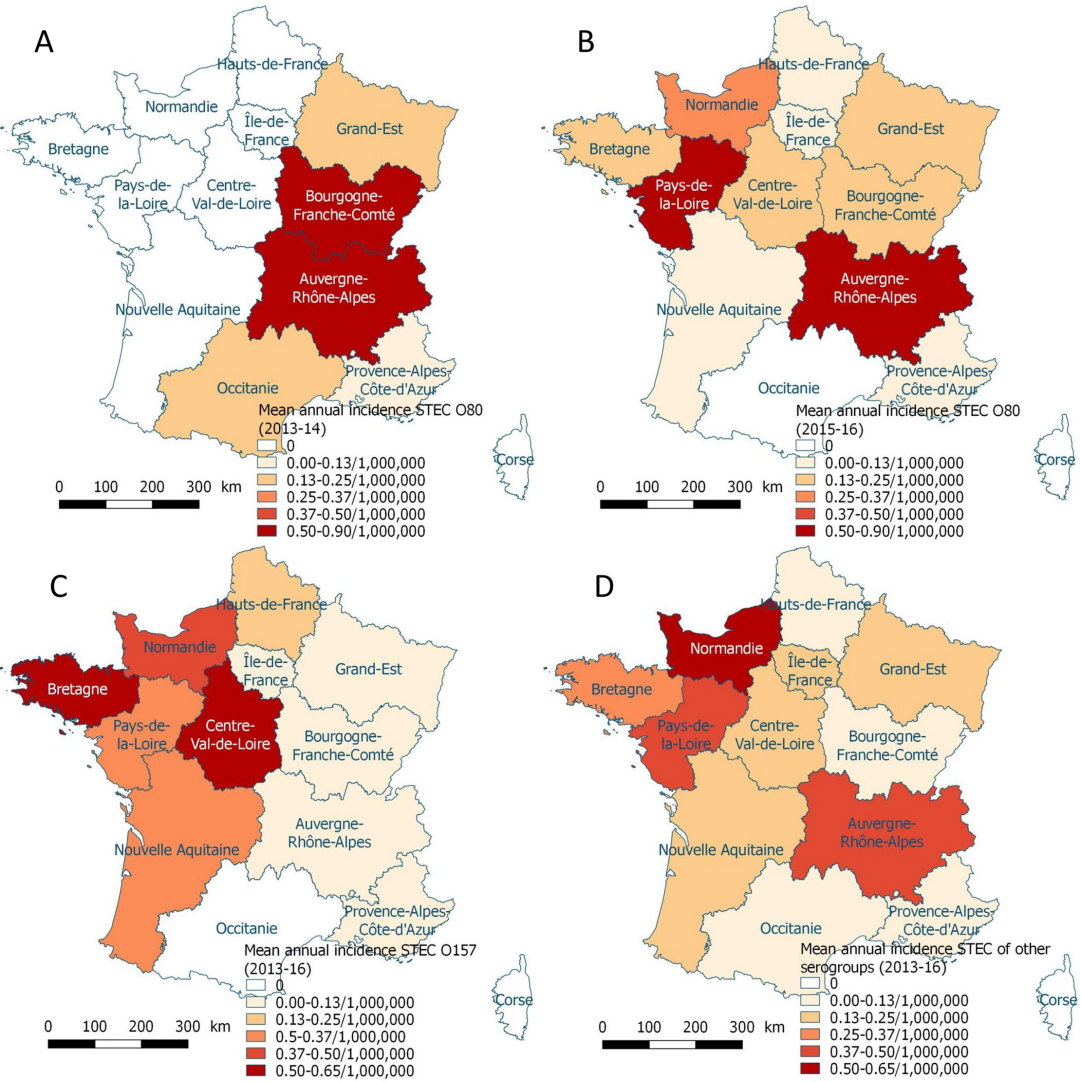
Mean annual incidence of STEC associated HUS in children per region in mainland France (A) of serogroup O80 in 2013–14; (B) of serogroup O80 in 2015–16; (C) of serogroup O157 in 2013–16; (D) of other non-O80 non-O157 serogroups in 2013–16.

In 2013/14, STEC O80 cases were geographically limited to the East of France (Fig 1A, S1 Table). From 2014 to 2016, we observed spread to all regions of mainland France (Fig 1B). STEC O157 was reported mostly in the West (Fig 1C). STEC of other serogroups were identified in all regions of mainland France (Fig 1D). 70% of STEC O80 cases lived in an urban municipality.

**Fig 2:**
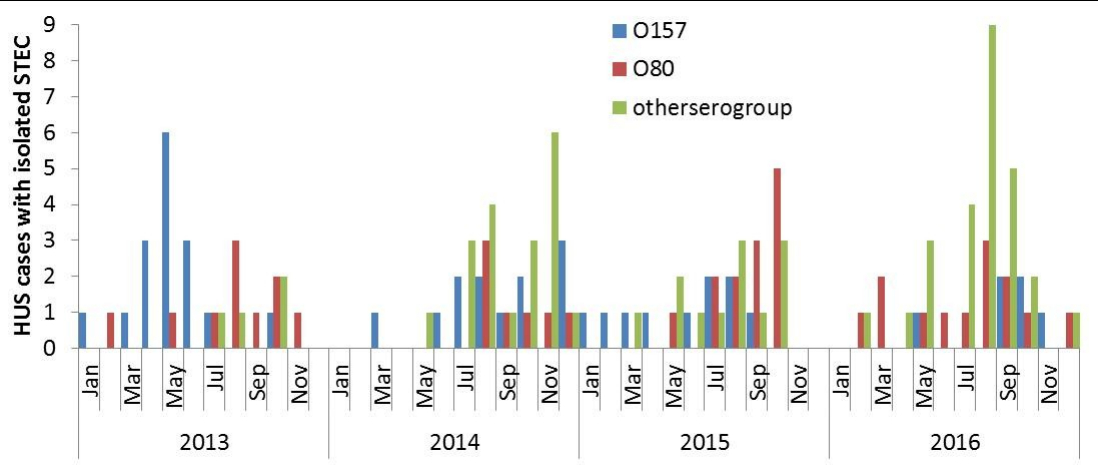
Monthly distribution of STEC infected HUS cases per serogroup reported in France 2013–2016.

Between July and October each year of 2013–2016, 72% (n = 31) of the STEC O80 cases, 41% (n = 18) of the STEC O157 cases and 70% (n = 43) of the STEC cases of other serogroups were reported. While O80 and other non O157 serogroups peaked during the summer months, STEC O157 cases were reported throughout the year, with peaks in the number of cases in different seasons: May in 2013, in December in 2014, July/August in 2015, and September/October in 2016 (Fig 2). We identified a twelve month periodicity in STEC O80 occurrence, with timing of the seasonal peak in the summer months, significantly different from the timing of STEC O157 peak incidence (p = 0.04).

The median age of STEC O80 infected pediatric HUS patients was 1.1 years (interquartile range (IQR) 0.7–1.8), significantly lower than the median age of STEC O157 infected patients (4.0 years; IQR 2.3–6.6; p<0.01) and patients infected with STEC of other serogroups (1.8 years; IQR 1.0–4.3; p<0.01). No STEC O80 infected patient was older than 6 years of age. Nineteen (43%) STEC O80, 25 (54%) STEC O157 and 23 (37%) STEC other serogroup infected patients were male, but these differences were not statistically significant (p = 0.29 and p = 0.53 respectively; Table 1).

**Table 1:** Comparison of characteristics of Shiga-toxin-producing *Escherichia coli* (STEC) O80 infected patients and of STEC O157 infected patients, and of patients infected with other STEC serogroups, pediatric HUS cases reported in France, 2013–16.

		O80		O157		p-value	Other serogroups		p-value
		n/N	%	n	%		n/N	%	
**Age group**	< 1 y	21/45	47	2/46	4	<0.01*	15/62	24	<0.01
	1–2 y	15/45	33	7/46	15		18/62	29	
	2–5 y	8/45	18	19/46	41		14/62	23	
	5-15y	1/45	2	18/46	39		15/62	24	
**Sex**	Male	19/44	43	25/46	54	0.29	23/62	37	0.53
**Housing**	House	6/11	55	11/15	73	0.42	14/19	74	0.43
	Apartment	5/11	45	4/15	27		5/19	26	

*Chi2 test of the age distribution

All cases met the surveillance criteria for acute hemolytic anemia: Mean hemoglobin among STEC O80 cases was 6.0 g/100mL (IQR 5.4–7.6), among STEC O157 cases 8.4 g/100mL (IQR 7.0–9.3), and among cases of other STEC serogroups 6.7 g/100mL (IQR 5.8–9.2); red blood cell fragments ≥ 2% were identified among 40 (89%) STEC O80 cases, 36 (78%) STEC O157 cases, and 55 (89%) cases of other STEC serogroups. For 21 (53%) STEC O80 cases, 3 (7%) STEC O157 cases and 16 (27%) STEC cases of other serogroups recorded creatinine levels were below the threshold for acute renal failure. Median creatinine levels at the time of reporting in patients below 2 years of age were 51 μmol/L (IQR 38–105) among STEC O80, 256 μmol/L (IQR 123–383; p = 0.03) among STEC O157 and 108 μmol/L (IQR 42–258; p = 0.11) among patients infected with STEC of other serogroups. In patients aged ≥2 years, median creatinine levels were 160 μmol/L (IQR 69–501) among STEC O80, 343 μmol/L (IQR 204–436; p = 0.36) among STEC O157 and 322 μmol/L (IQR 199–673; p = 0.26) among patients infected with STEC of other serogroups. Diarrhea before HUS was reported by 35 (80%) of STEC O80 cases, significantly lower than 44 (100%) of STEC O157 cases (p<0.01), but not significantly different from 54 (89%) of cases of other STEC serogroups (p = 0.27). The median duration of diarrhea was 6 days (IQR 4–16; n = 3) among STEC O80 cases, compared to 6 days (IQR 5–7; n = 4) among STEC O157 cases (p = 0.86), and to 7 days (IQR 6–7; n = 10) among cases of other STEC serogroups (p = 0.79).

STEC O80 cases were as likely as STEC O157 cases or STEC cases of other serogroups to attend nursery or school, to live in an individual house, to have swum in a pool, lake, pond or the sea, or to have been in contact with farm or pet animals (Table 2).

**Table 2:** Comparison of characteristics and exposures of Shiga-toxin-producing *Escherichia coli* (STEC) O80 infections to STEC O157 infections, and to STEC infections of other non O80 non O157 serogroups, among pediatric HUS cases reported in France, 2013–16.

		O80		O157		O80 compared to O157		Other serogroups		O80 compared to other serogroup	
		n/N	%	n/N	%	OR (95% CI)	aOR (95% CI)*	n/N	%	OR (95% CI)	aOR (95% CI)*
**Living next to**	A park	5/10	50	2/12	14	6.0 (0.64–77)		3/17	18	4.67 (0.60–40)	
	A wood	6/10	60	2/15	13	9.8 (1.0–122)		6/18	33	3.00 (0.47–20)	
	Cultivated land	4/9	44	5/14	36	1.4 (0.19–11)		3/15	17	4.00 (0.46–36)	
	Grazing land	3/10	30	7/15	47	0.49 (0.06–3.4)		3/18	17	2.14 (0.22–19)	
**Living in urban municipality**		31/44	70	26/46	57	1.8 (0.71–4.8)		30/59	51	2.31 (0.94–5.8)	3.4 (0.99–11)
**Contact with a person with diarrhea or HUS**		8/38	21	20/41	49	0.28 (0.09–0.83)	0.13 (0.02–0.78)	25/55	45	0.32 (0.11–0.89)	0.14 (0.04–0.51)
**Attending nursery or school**		21/41	51	26/41	63	0.61 (0.23–1.6)		29/50	58	0.76 (0.31–1.9)	
**Contact with animals**	Farm	11/39	28	10/39	26	1.1 (0.37–3.5)		15/54	28	1.0 (0.36–2.8)	
	Pets	4/11	36	8/16	50	0.57 (0.09–3.5)		12/19	63	0.33 (0.05–2.0)	
	Wild	0/10		3/15	20			0/17	0		
**Swimming**	Any	11/41	27	8/42	19	1.56 (0.49–5.1)		14/53	26	1.0 (0.36–2.8)	
	Pool	3/12	25	1/15	7	4.76 (0.37–50)		5/19	26	0.93 (0.17–5.0)	
	Pond, lake or sea	0/12	0	0/15	0			2/16	11		
**Food consumption**	Ground beef steak	11/36	31	30/37	81	0.10 (0.03–0.34)	0.14 (0.02–0.80)	29/49	59	0.30 (0.11–0.82)	0.58 (0.16–2.1)
	Any cooked meat	24/28	86	36/38	95	0.33 (0.03–2.6)		43/45	96	0.28 (0.02–2.1)	
	Any processed meat product	17/22	77	25/26	96	0.14 (0.00–1.4)		27/32	84	0.63 (0.13–3.2)	
	Raw cow milk	3/38	8	3/40	8	1.06 (0.13–8.4)		2/50	4	2.1 (0.22–25)	
	Cheese from raw cow milk	10/37	27	13/40	33	0.77 (0.25–2.3)		12/49	24	1.1 (0.38–3.4)	
	Raw vegetables	12/22	55	21/27	78	0.34 (0.08–1.4)		25/30	83	0.24 (0.05–1.0)	0.08 (0.01–1.2)
**Water consumption**	Untreated (from well or fountain)	1/19	5	2/26	8	0.67 (0.01–14)		1/24	4	1.3 (0.02–105)	

OR, crude odds ratio; aOR, adjusted odds ratio; 95% CI, 95% confidence interval; HUS, hemolytic and uremic syndrome

* Adjusted for age and exposures that were collected for all study patients and that were retained in the multivariable model. aOR for exposures retained in the final model are presented in the table.

Among those for whom the information was available, STEC O80 cases (60%) were more likely to live next to a wood than STEC O157 cases (13%; OR 9.8 95%CI 1.0–122). We did not find STEC O157 cases or cases of other serogroups to be more likely to live next to cultivated (OR 1.4 95%CI 0.19–11 and 4.00 95%CI 0.46–36 respectively) or grazing lands (OR 0.49 95%CI 0.06–3.4 and 2.14 95%CI 0.22–19 respectively) than those with STEC O80 infection.

In multivariable analysis, compared with STEC O157 cases, STEC O80 cases were less likely to have consumed ground beef steak (aOR 0.14 95% CI 0.02–0.80) and have had contact with a person presenting with diarrhea or HUS (aOR 0.13 95%CI 0.02–0.78). Compared with STEC cases of other serogroups, STEC O80 cases were also less likely to have had contact with a person with diarrhea or HUS (aOR 0.14 95%CI 0.04–0.51) but were more likely to live in an urban environment (70% vs 51%; aOR 3.40 95%CI 0.99–11).

## Discussion

STEC O80 spread from the East to all regions of France during 2013–2016. Our study identified several differences between STEC O80 associated HUS and HUS associated with other STEC serogroups among children in France. STEC O80 infected children were significantly younger and less likely to have eaten ground beef than STEC O157 infected children. Furthermore, STEC O80 infected children were more likely to live in an urban municipality than children infected other STEC serogroups and more often in proximity of woods than STEC O157 infected children. They were less likely to have been in contact with a person with diarrhea or HUS, suggesting person-to-person transmission could be less important. Lower occurrence of diarrhea among STEC O80 cases could partially explain why contact with diarrhea cases may have been more frequent among STEC O157 cases. However, STEC O157 infected patients were 8 times more likely to have been in contact with a person with diarrhea or HUS than STEC O80 infected patients, so probably there are more important underlying reasons than just this difference in occurrence of diarrhea.

Consumption of ground beef was less associated with STEC O80 infection than with STEC O157 and other O serogroups, suggesting that the relationship between STEC infection in humans and the cattle reservoir is potentially less important among STEC O80 infections than among STEC O157. Nevertheless, we did not find any difference in contact with farm animals between STEC O80 and O157 cases. Consumption of ground beef and living in areas with higher cattle density have previously been identified as key risk factors for STEC O157, which has cattle as its main reservoir [4,13,14]. Enteropathogenic *E*. *coli* O80 with similarities to human STEC O80 has been found in calves [15]. However, studies reported no significant association between cattle density and STEC human infection with other STEC serogroups, suggesting that not all serogroups necessarily share the same reservoirs [5,14]. This is consistent with our study that suggested differences in potential risk factors, and possibly in reservoir, between STEC O80 and O157 infections. Further case-control or ecological studies may be needed to further identify specific risk factors for STEC O80 infections.

The geographic distribution of STEC O80 from the East of France in 2013/14 to most of mainland France in the following years is different from the STEC O157 distribution observed mainly in the West of France throughout the study period (Fig 1A–1D, S1 Table). The urban distribution of STEC O80 cases (70% urban) reflects that of the general population in France (78%). In contrast, other STEC serogroups were more frequently reported from rural areas. With most STEC O80 cases reported between August and October, similarly to STEC O80 infections reported in Switzerland, the seasonal pattern is nevertheless not very different from what we observed for other STEC serogroups [5,11].

At the time of reporting, serum creatinine levels were below the minimum threshold for acute renal failure in about half of STEC O80 cases and a fourth of STEC cases of other serogroups. Therefore, these cases did not strictly fulfill the criteria of the HUS case definition used for surveillance. Acute hemolytic anemia, the second condition of the case definition, was nevertheless identified among all study cases. Considering the increasing importance of STEC serogroups other than O157, the current HUS surveillance case definition may need to be revised.

Study limitations were related to the study design and the data we used. First, our study included only pediatric HUS cases, excluding adults and milder cases of STEC infection. These cases constitute a fraction of all STEC infections and may not be representative of all STEC infections in France. Whereas our study population was limited to children with HUS, a previous study on STEC O80 in France, not limited to pediatric HUS, found only one adult case over a 10 years study period [10]. Second, using a case-case study design does not allow identifying risk factors common to all serogroups, or could underestimate an association with a potential factor if the “control” cases are more likely to be exposed to that factor than the source population. By using two different comparison groups, we avoided that an association with a factor known to be associated with STEC O157 but not with STEC of other serogroups would remain undetected. In addition, in the case-case study design, the comparison groups underwent the same selection process of diagnosis and reporting, and should not have more difficulties to recall exposures, while differences in an exposure of interest can be revealed [16]. Third, several possible exposures of interest were not recorded for all cases. Analyses of such exposures may have lacked power to identify a significant association. In the multivariable regression model, only variables recorded for all cases could be included.

## Conclusion

During 2013–2016, STEC O80 infections spread among young children with HUS all over mainland France. Our study indicated differences between STEC O80 and STEC O157 or other serogroup infections in: i) potential risk factors suggesting the existence of different reservoirs, ii) transmission patterns, iii) age and geographical distribution. To identify the potential reservoirs and sources of infection a better understanding of the environment to which STEC O80 infected young children are exposed is needed. Case investigations should routinely include extensive questionnaires, with more detail on potential environmental exposures. Studies isolating STEC in animals could elucidate the search for a reservoir at the origin of STEC O80 infection in children.

## Supporting information

S1 Table Comparison of the number of Shiga-toxin-producing *Escherichia coli* (STEC) O80 and O157 infections reported between three Eastern regions of France* and other regions of mainland France in 2013/14 and 2015/16, pediatric HUS cases reported in France, 2013–16. (DOCX)
