# Supplementary material for: Emerging Shiga-toxin-producing Escherichia coli serogroup O80 associated hemolytic and uremic syndrome in France, 2013-2016: Differences with other serogroups
Source: PLoS One. 2018 Nov 12;13(11):e0207492. doi: 10.1371/journal.pone.0207492 (PMC6231688; doi:10.1371/journal.pone.0207492)
Supplement: S1 Table — (DOCX) [file pone.0207492.s001.docx]

**S1 Table. Comparison of the number of Shiga-toxin-producing *Escherichia coli* (STEC) O80 and O157 infections reported between three Eastern regions of France* and other regions of mainland France in 2013/14 and 2015/16, pediatric HUS cases reported in France, 2013-16.**

| STEC serogroup | Years | Eastern regions | | Other regions | | p-value** |
| --- | --- | --- | --- | --- | --- | --- |
|  |  | n | % | n | % |  |
| O80 | 2013/14 | 16 | 84 | 3 | 16 | 0.01 |
|  | 2015/16 | 12 | 46 | 14 | 54 |  |
| O157 | 2013/14 | 7 | 32 | 15 | 68 | 1.00 |
|  | 2015/16 | 12 | 32 | 25 | 68 |  |

HUS, hemolytic and uremic syndrome; * Auvergne-Rhône-Alpes, Grand-Est and Bourgogne Franche-Comté regions ; ** Fisher's exact test
